# Nafamostat Mesylate Regulates Glycosylation to Alleviate Aristolochic Acid Induced Kidney Injury

**DOI:** 10.3390/toxins17030145

**Published:** 2025-03-18

**Authors:** Pei Xie, Huijun Liu, Xingli Huo, Junlong Chen, Yu Li, Yu Huang, Zongning Yin

**Affiliations:** 1 Key Laboratory of Drug-Targeting and Drug Delivery System of the Education Ministry, Drug Targeting and Drug Delivery System Key Laboratory of Sichuan Province, Sichuan Engineering Laboratory for Plant-Sourced Drug, Sichuan Research Center for Drug Precision Industrial Technology, West China School of Pharmacy, Sichuan University, Chengdu 610041, China; xie6620@163.com (P.X.); liuhuijunhx@163.com (H.L.); xinglihuo@163.com (X.H.); chenjunlong0413@163.com (J.C.); ly09020805@163.com (Y.L.); 2 Co-Construction Collaborative Innovation Center for Chinese Medicine Resources Industrialization by Shaanxi & Education Ministry, Shaanxi University of Chinese Medicine, Xianyang 712046, China; 3 Haisco Pharmaceutical Group Co., Ltd., Chengdu 611130, China

**Keywords:** nafamostat mesylate, acute kidney injury, glycosylation, protein folding, aristolochic acid

## Abstract

Acute kidney injury (AKI) is a condition with a poor prognosis, exacerbated by the lack of effective therapeutic options and inadequately understood underlying mechanisms. Glycosylation, a post-translational modification of proteins, is essential for maintaining protein stability and function, and its dysregulation leads to protein misfolding and amyloid aggregation. Glycosylation dynamics are implicated in several pathologies, including inflammation, cancer, and AKI, highlighting the therapeutic potential of regulating glycosylation and preventing aggregation in AKI treatment. This study investigates the effect of nafamostat mesylate (NM) on protein glycosylation and amyloid aggregation in vivo. Using optical spectroscopy and other analytical techniques, we demonstrate that NM restores glycosylation levels and inhibits protein aggregation in aristolochic-acid-induced acute kidney injury. The mechanism likely involves enzymatic modulation that corrects hypoglycosylation and prevents amyloid aggregation, promoting proper protein folding and enhancing its stability. These findings suggest that NM may provide a novel therapeutic strategy for AKI and other glycosylation-related diseases, underscoring the potential for early intervention and treatment of these conditions.

## 1. Introduction

Acute kidney injury (AKI) is a complex, multifactorial syndrome that manifests as a continuum of disease, ranging from mild renal impairment to complete renal failure. Renal failure represents the severe stage of its progression, and its outcome is influenced by various factors, including underlying diseases, the degree of injury, and its duration [1,2,3], characterized by renal tubular damage, interstitial inflammation, and a rapid decline in kidney function [4,5]. The incidence and mortality rates of AKI are alarmingly high, particularly in high-risk patients in intensive care units, where it has emerged as a major challenge within global healthcare systems [6,7,8]. If left untreated, severe or recurrent episodes of AKI may progress to chronic kidney disease (CKD) or end-stage renal disease (ESRD) [9,10,11], underscoring the critical importance of early intervention. Despite significant advances in understanding the pathological mechanisms underlying AKI in recent years, current treatments remain limited to symptomatic support, with a dearth of pharmacological strategies targeting its underlying causes [12]. Therefore, the identification of novel therapeutic agents that can intervene early in the disease process is of paramount importance. In this regard, serine protease inhibitors have gained attention as a promising therapeutic strategy for kidney injury induced by various etiologies [13,14].

Nafamostat mesylate (NM) is a potent, broad-spectrum serine protease inhibitor with established efficacy in the treatment of conditions such as pancreatitis and disseminated intravascular coagulation [15,16,17]. As research into its multi-target properties expands, NM has shown promise in treating liver failure [18], providing neuroprotection [19,20], and in oncology [21,22]. In the context of kidney disease, studies have demonstrated that NM significantly improves outcomes in patients with end-stage renal failure [23]. Notably, NM has been shown to enhance the endothelial glycosaminoglycan structure in diabetic mice, alleviate renal tubular damage, and inhibit apoptosis, thereby offering protection against ischemia/reperfusion injury [24,25,26]. However, the precise mechanisms by which NM exerts its effects in AKI remain incompletely understood.

Recent research suggests that the onset of AKI is closely linked to protein misfolding, endoplasmic reticulum (ER) stress, and associated post-translational modifications [27]. ER function is crucial for maintaining protein homeostasis, and renal tubular cell responses to ER stress are pivotal in preserving normal kidney function [28]. Glycosylation, the most prevalent form of protein post-translational modification, primarily occurs in the ER. This modification is essential not only for ensuring proper protein folding but also for maintaining their functional integrity [29,30,31,32,33]. Alterations in glycosylation have been implicated in the progression of immune-mediated nephritis and chronic kidney disease [34,35]. However, systematic studies investigating changes in protein glycosylation and their mechanisms in AKI remain limited.

In this context, this study aims to explore the therapeutic potential of NM in AKI, specifically focusing on how it modulates protein glycosylation levels and prevents the aggregation of misfolded proteins to repair renal damage. We hypothesize NM can restore protein conformation in the early stages of AKI by modulating glycosylation levels, thus promoting the recovery of renal tubular cell function. Compared to traditional therapies, early intervention with NM in AKI may offer improved outcomes, delay disease progression, and reduce the risk of developing CKD.

This study employs aristolochic acid nephropathy (AAN) as an experimental model. Aristolochic acids (AAs) are highly nephrotoxic compounds [36,37]. AAN has become a focal point in nephrology, particularly regarding the mechanisms underlying the transition of AKI caused by AAs to chronic kidney disease [38]. Moreover, the zebrafish model provides a unique opportunity to observe the dynamic changes in kidney injury and glycosylation associated with AAN, offering valuable insights into NM’s therapeutic effects through glycosylation modulation.

## 2. Results 

### 2.1. NM Alleviates Oxidative Damage in HK-2 Cells and Reduces Renal Tissue Injury in Zebrafish

**Figure 1:**
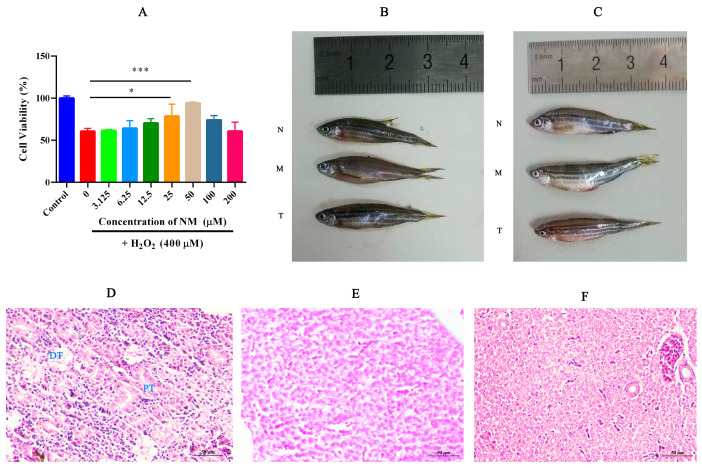
(**A**) The cell viability of HK-2 cells under oxidative stress after NM treatment, (*** *p* < 0.001,* *p* < 0.05). (**B**,**C**) The appearance images of male and female zebrafish (N: normal group, M: model group, and T: treatment group). (**D**–**F**) HE-stained kidney sections from zebrafish in the normal, treatment, and model groups, respectively. Scale bar: 50 μm; PT: proximal tubule; DT: distal tubule.

The protective effects of NM on HK-2 cells were evaluated across a concentration range of 6.25 to 100 μM (Figure 1A). The optimal concentration was determined to be 50 μM, at which the cell survival rate was restored to approximately 96%, suggesting a strong reparative capacity. This effect may be attributed to increased drug uptake at higher concentrations, which potentiates the antioxidant and reparative actions. Moreover, elevated drug concentrations appear to enhance membrane permeability in damaged cells, thereby further improving its therapeutic efficacy. These findings emphasize the importance of identifying the optimal dosage for maximizing therapeutic outcomes.

Acute kidney injury was successfully induced by AAs, as evidenced by Figure 1B,C (B for males; C for females), which exhibited typical pathological features, including abdominal distension, glossy abdominal skin, and increased mucus secretion on the body surface. Following NM treatment, these pathological changes were significantly alleviated, indicating that NM can mitigate kidney injury caused by AAs and promote the restoration of normal kidney function. H&E staining demonstrated the pathological changes in the kidney. The kidney tissue of the normal group zebrafish shows normal renal tubule morphology, with smooth and intact brush borders and basal membranes (Figure 1D), where pathological changes such as loss of the renal brush border, cell shedding, disorganization of the basement membrane, and vacuolization were observed (Figure 1F). NM treatment reduced pathological damage in the model, showing a trend towards recovery, particularly in the reduction in vacuolization (Figure 1E). This further supports the potential of NM for kidney repair in zebrafish.

The in vitro and in vivo results highlight the significant cell protective and repair effects of NM. Whether these effects are linked to glycosylation levels and protein functionality remains to be further investigated.

### 2.2. Glycosylation Degree Characterization

**Figure 2:**
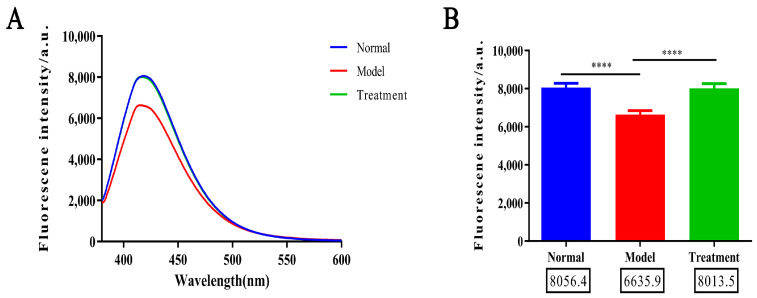
(**A**) The glycosylation fluorescence spectra for different groups. (**B**) Comparison in the fluorescence intensities across the groups (**** *p* < 0.0001).

As shown in Figure 2A,B, the fluorescence intensity of AGEs in the model group was significantly reduced compared to the normal group, suggesting that, following the induction of the AAN model, glycosylation levels in the animals are diminished, and AGEs production is consequently decreased. This reduction may be attributed to a decrease in the activity of glycosylation-related enzymes, resulting in impaired glycosylation reactions and subsequent protein functional damage, which disrupts biological activity and normal physiological function. In contrast, the fluorescence intensity of AGEs in both the normal and NM treatment groups increased significantly under excitation at 420 nm, indicating that glycosylation levels in these groups were higher. The fluorescence curves of the treatment and normal groups closely overlapped, suggesting that NM can modulate the glycosylation process, promoting AGEs production and restoring normal glycosylation levels. This effect may be mediated through mechanisms such as enhancing the activity of glycosylation enzymes, inhibiting AGEs degradation, or maintaining intracellular glycosylation homeostasis, ultimately helping to restore protein function and alleviate pathological damage.

### 2.3. Effect of NM on Protein Conformation in the Glycosylation System

We next examine the reparative effects of NM on protein structural alterations in the AAN model, employing endogenous fluorescence spectroscopy, synchronous fluorescence spectroscopy, and CD spectroscopy.

**Figure 3:**
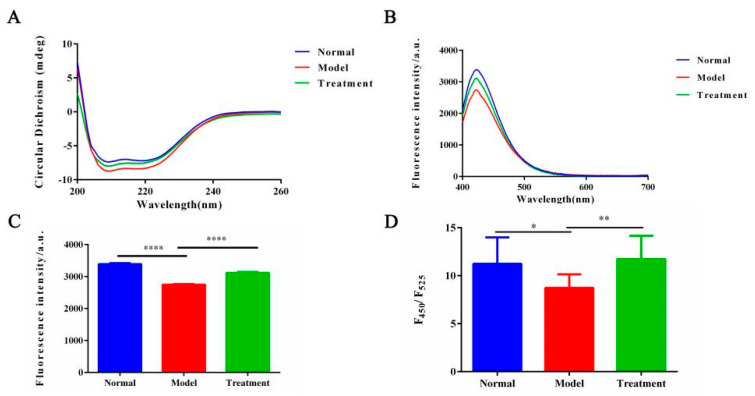
(**A**) The CD spectra of proteins for different groups. (**B**) The ANS fluorescence spectra from different groups. (**C**) Comparison in the ANS fluorescence intensities across the groups, (**** *p* < 0.0001). (**D**) The F_450_/F_525_ fluorescence ratio from the ANS spectra across the groups, (** *p* < 0.01, * *p* < 0.05).

CD spectroscopy is a powerful technique for analyzing protein secondary structures and monitoring their dynamic changes [39]. As shown in Figure 3A, CD spectral analysis showed that the proteins in the normal group displayed negative peaks at 208 nm and 222 nm, indicating a predominance of α-helix structure [40], with the width of the 222 nm negative peak reflecting high structural stability. The spectra of the treatment group closely resembled those of the normal group, with the depth and width of the 222 nm negative peak being nearly identical, suggesting that NM treatment significantly restored the α-helix structure of the proteins. In contrast, the model group exhibited a marked reduction in the negative ellipticity at 222 nm, with the curve becoming flatter, indicating a decrease in α-helix content. Additionally, the positive peak at 213 nm disappeared, possibly reflecting an abnormal accumulation of non-native β-sheet structures, accompanied by the formation of protein aggregates. These abnormal changes are likely due to protein misfolding caused by reduced glycosylation levels. The CD results further confirm that NM, by regulating glycosylation levels, promotes proper protein folding and restores the normal protein conformation. CD spectral analysis indicates that, in the AAN model, the α-helix structure is significantly disrupted, with an increase in β-sheet structures, which is strongly associated with protein functional loss. In contrast, the treatment group showed significant restoration of protein secondary structure, suggesting that NM intervention may play a key role in structural restoration and functional recovery.

**Figure 4:**
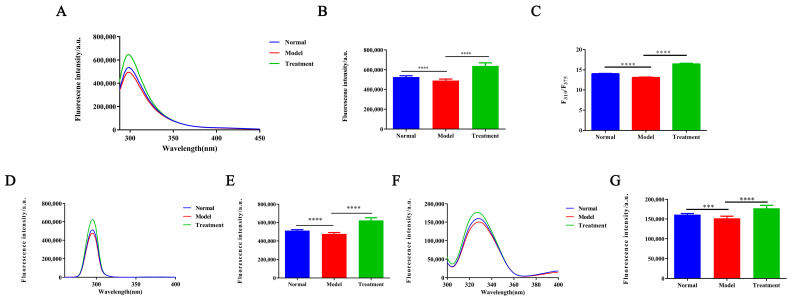
(**A**)The endogenous fluorescence spectra from different groups. (**B**) Comparison in the endogenous fluorescence intensities across the groups. (**C**) The F_310_/F_375_ ratio from the endogenous fluorescence spectra. (**D**) The tyrosine fluorescence spectra. (**E**) The tyrosine fluorescence intensities. (**F**) The tryptophan fluorescence spectra. (**G**) The tryptophan fluorescence intensities, (**** *p* < 0.0001, *** *p* < 0.01).

Figure 4A,B demonstrate that glycosylation levels are significantly reduced in the AAN model group, resulting in a decrease in endogenous fluorescence intensity. In contrast, the NM treatment group exhibits a marked recovery in fluorescence intensity, indicating effective restoration of glycosylation levels and subsequent improvement in protein structure. The F_310_/F_375_ ratio and peak shift serve as quantitative indicators of protein conformational changes due to glycosylation. Figure 4C shows that the model group has a significant reduction in the F_310_/F_375_ ratio (*p* < 0.0001) and a red shift in the fluorescence spectrum, suggesting that insufficient glycosylation modifications lead to alterations in the aromatic amino acid microenvironment. These alterations result in protein conformational instability or misfolding, thereby affecting fluorescence properties. In the NM treatment group, the F_310_/F_375_ ratio significantly increased (*p* < 0.0001), suggesting that NM treatment may restore glycosylation by modulating glycosylating enzyme activity or regulating inhibitory factors [41,42], allowing the protein to achieve proper modification. Following glycosylation recovery, the fluorescence spectrum returned to normal, and the increase in the F_310_/F_375_ ratio reflected the transition of the protein from misfolded to its native conformation. As shown in Figure 4D–G, fluorescence intensities of tyrosine and tryptophan were reduced after modeling. However, after NM treatment, fluorescence intensities of both amino acids significantly increased, consistent with steady-state experimental findings.

The ANS fluorescence spectrum combined with the F_450_/F_525_ ratio reveals protein conformation and the exposure of hydrophobic regions. In Figure 3D, the model group exhibits a decrease in the F_450_/F_525_ ratio, indicating a reduction in hydrophobic region binding with ANS. The hydrophobic regions are sequestered in non-native structures, forming stable aggregates that shield the hydrophobic regions, reducing the exposure of ANS binding sites and decreasing fluorescence intensity. After NM treatment, the F_450_/F_525_ ratio significantly increased, reflecting recovery of protein conformation and increased exposure of hydrophobic regions. This suggests that NM treatment inhibits aggregation formed by hydrophobic region interactions, thereby reducing shielding of these regions. This further supports the ability of NM to regulate protein conformation, prevent misfolding, and reduce aggregate formation.

Taken together, these results, including endogenous fluorescence spectra, ANS fluorescence spectra, and synchronous fluorescence analysis, confirm that NM can restore the abnormal protein conformation caused by glycosylation level disturbances in the AAN model. NM treatment not only restored normal glycosylation levels by increasing the fluorescence intensity of AGEs but also improved protein function by repairing protein folding and stabilizing its structure. The therapeutic potential of NM in kidney injury repair, particularly in regulating glycosylation and protein conformation, provides important theoretical and experimental evidence for the future treatment of related diseases.

### 2.4. The Effect of NM on Glycosylation-Induced Protein Aggregation

We next investigate the impact of NM on misfolded proteins and its role in inhibiting protein aggregation through multiple experimental approaches, with a focus on AA-induced aggregation.

**Figure 5:**
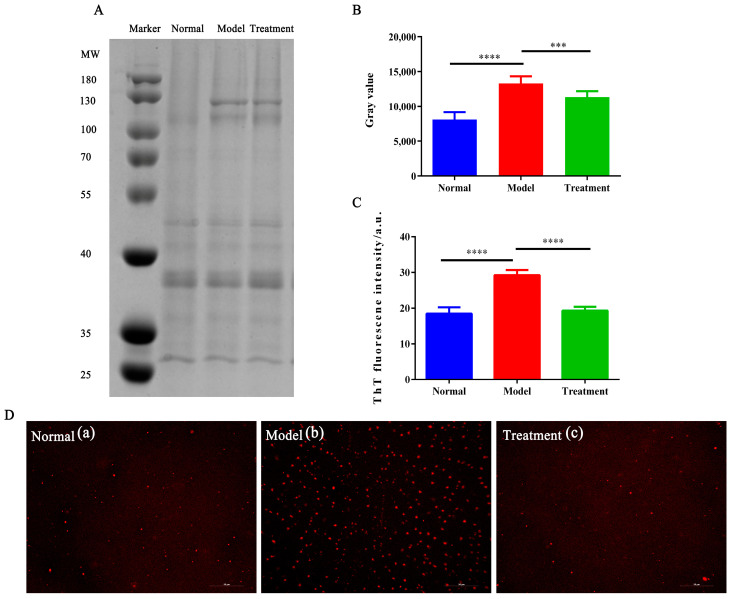
The protein electrophoresis band patterns (**A**). The grayscale analysis of the electrophoresis results (**B**). The ThT fluorescence intensity (**C**). Nile-red-stained protein aggregates observed under an inverted fluorescence microscope (**D**), a: Normal group, b: model group, c: treatment group, scale bar: 50 μm, (**** *p* < 0.0001, *** *p* < 0.01).

As shown in Figure 5A, protein aggregation in the 70–100 kDa region was significantly increased in the model group (intensity: 13270.3) compared to the normal group (intensity: 8114.5, *p* < 0.0001), indicating enhanced aggregation due to AA induction. After NM treatment, the intensity in the experimental group decreased to 11322.6 (*p* < 0.001), indicating reduced aggregation and partial restoration of protein stability.

ThT, a probe known for specifically binding to β-sheet structures [43], demonstrated an increased fluorescence signal in the model group, reflecting the transition of proteins from their native conformation to misfolded β-sheet aggregates under AA induction. In contrast, the fluorescence intensity was significantly reduced in the treatment group following NM intervention, indicating the inhibition of β-sheet aggregation and a protective effect of NM on protein folding (Figure 5B,C).

As shown in Figure 5D, Nile Red binds to hydrophobic regions of proteins and revealed a dense and enlarged distribution of protein aggregates in the model group, suggesting significant misfolding induced by AA. In the NM-treated group, the number and distribution of aggregates were notably improved, indicating that NM effectively inhibited aggregation and helped restore the native protein conformation.

### 2.5. Fluorescence Quenching of Protein Interactions with NM

**Figure 6:**
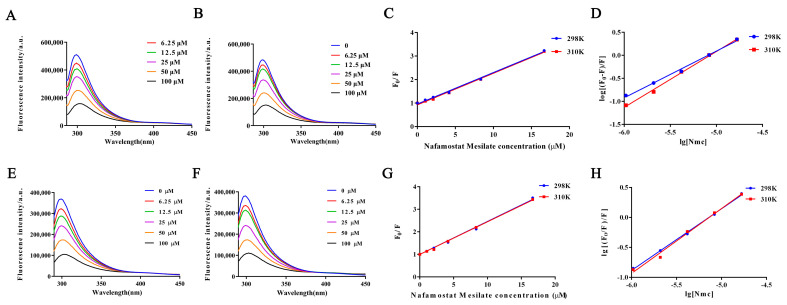
Interactions between proteins in the normal animal group and NM at 298 K (**A**) and 310 K (**B**); Stern Volmer (**C**) and Scatchard (**D**) equations for the interaction between normal histones and different concentrations of NM at 298 K and 310 K; the interaction maps of proteins in the model group animals with NM at 298 K (**E**) and 310 K (**F**); Stern Volmer (**C**) and Scatchard (**D**) equations for the interaction between model histones and different concentrations of NM at 298 K and 310 K; Stern-Volmer equation (**G**) and Scatchard equation (**H**) for the interaction of the protein with different concentrations of NM at 298 K and 310 K.

Previous experiments confirm that NM can inhibit protein aggregation by restoring glycosylation levels. Next, we further investigated the interaction of NM with proteins. Figure 6A,B,E,F illustrate the fluorescence spectral changes in zebrafish proteins upon NM treatment at 25 °C and 37 °C. These data suggest that NM may interact with proteins through either static or dynamic mechanisms, potentially forming complexes or modifying the protein microenvironment.

Collectively, the fluorescence quenching experiments demonstrate that NM not only binds to proteins with high affinity but also modulates their conformation and functional state. These results offer critical insights into the mechanistic basis of NM’s role in protein regulation.

Table 1 shows the results of the quenching constant (K*_SV_*) and quenching rate constant (Kq) calculated using the Stern–Volmer equation for proteins in the normal group and model group at different temperatures. In the model group, K*_SV_* decreased with increasing temperature, suggesting that NM primarily quenches fluorescence of proteins in zebrafish through static quenching. This observation aligns with the characteristic that the stability of the protein–NM complex decreases with temperature. Furthermore, the calculated Kq is significantly greater than the upper limit of the fluorescence lifetime for biomacromolecules, further supporting the static quenching mechanism.

Thermodynamic analysis reveals that, in the normal group, Δ*H* > 0, Δ*S* > 0, and Δ*G* < 0, indicating that the binding of NM to proteins is an endothermic process primarily driven by hydrophobic interactions. In contrast, in the AAN model group, Δ*H* < 0, Δ*S* > 0, and Δ*G* < 0, suggesting that binding is exothermic, mainly governed by hydrogen bonds and van der Waals forces. This thermodynamic difference reflects significant changes in protein conformation and the microenvironment between the normal and model groups.

Combined with the Stern–Volmer and thermodynamic analyses, these findings indicate that NM binds to zebrafish proteins through static quenching. In the AAN model, the binding characteristics and thermodynamic changes suggest that NM effectively modulates protein conformation, reducing aggregation caused by misfolded proteins. This study provides solid experimental evidence and theoretical support for the potential therapeutic mechanism of NM in treating AAN.

## 3. Conclusions

In this study, we induced an acute kidney injury model in zebrafish, a widely used model organism for human disease research [44,45,46], to systematically investigate the role of NM in regulating glycosylation levels and inhibiting protein misfolding and amyloid aggregation. The experimental results demonstrate that NM significantly alleviates oxidative-stress-induced damage to renal tubular epithelial cells, restores glycosylation levels, prevents abnormal protein aggregation, and preserves protein structure. Fluorescence quenching experiments further confirmed the direct interaction between NM and proteins. Both in vivo and in vitro data substantiate the efficacy of NM in correcting glycosylation imbalance. These findings provide, for the first time, compelling evidence of NM in regulating glycosylation and maintaining protein homeostasis, offering both theoretical and experimental support for the treatment of AKI and other glycosylation imbalance-related diseases. NM has been used in clinical practice for end-stage renal disease and continuous kidney replacement therapy (CKRT) [47,48]. Notably, recent clinical research by Liu et al. has thoroughly demonstrated the advantages of NM in continuous renal replacement therapy (CRRT) for patients with a high risk of bleeding [49]. These results provide clinical evidence supporting NM’s new indications and further endorse its therapeutic potential in renal protection and the management of acute kidney injury. This study not only elucidates the molecular mechanism underlying the action of NM but also expands its potential applications in nephrology, providing novel treatment strategies for these conditions. Furthermore, our results underscore the substantial therapeutic potential of NM as a multifunctional agent for AKI, highlighting the innovation and significance of this research in advancing our understanding of disease mechanisms and exploring new therapeutic strategies.

## 4. Materials and Methods

### 4.1. Materials

BSA protein quantification kit (Wuhan Saiwei Biological Technology Co., Ltd., Wuhan, China), Aristolochic Acid A (Chengdu Naqi Lithium Biotechnology Co., Ltd., Chengdu, China), Nafamostat mesylate (prepared by West China School of Pharmacy, Sichuan University, Chengdu, China), D-Mannitol (Injection/M-109-7, Pfanstiehl, Waukegan, IL, USA), and Succinic Acid (Nanjing Chemical Reagents Co., Ltd., Nanjing, China) were used. Protein lysis buffer (Wuhan Saiwei Biological Technology Co., Ltd., Wuhan, China) and zebrafish (AB strain) were used.

### 4.2. Preparation of Test Sample

NM and D-Mannitol were weighed (1:2) and lyophilized with an appropriate amount of Succinic Acid to adjust pH. The lyophilized powder was reconstituted with 5% glucose and diluted with saline to the desired concentration. It was sterilized by 0.22 µm filtration.

### 4.3. Protective Effect of NM on Oxidative Damage in HK-2 Cells

HK-2 cells were cultured in DMEM with 15% FBS and 1% penicillin–streptomycin. Cells were seeded at 0.5 × 10^4^ cells/well in 96-well plates and treated with serial dilutions (200 to 3.125 μM). After 400 μM H_2_O_2_ induction for 24 h, MTT assay was used to measure cell viability as in Equation (1).

Cell viability (%) = (A_experimental_ − A_blank_)/(A_control_ − A_blank_) × 100% (1)

### 4.4. Animal Husbandry and Model Establishment

Adult zebrafish were housed for 2 weeks at 25–28.5 °C with a 14/10 h light/dark cycle. Groups: normal, model, and treatment (NM 50 ng, 5.0 µg/mL). The zebrafish were exposed to breeding water containing aristolochic acid (60 µmol/L) for five consecutive days to induce the renal injury model [50,51]. NM was administered daily (10 µL, intraperitoneal) for 5 days [52]. All procedures were approved by the Animal Ethics Committee of Sichuan University (KS2023512).

### 4.5. Renal Tissue Histology and Protein Extraction

#### 4.5.1. Zebrafish Anesthesia and Sample Collection

This study strictly follows the ethical standards for humane euthanasia of fish outlined in the AVMA Guidelines for the Euthanasia of Animals (AVMA 2020) and the European Parliament and Council Directive 2010/63/EU, ensuring that all experimental procedures comply with internationally recognized animal welfare and ethical requirements.

In the experiment, all adult zebrafish were fasted for 24 h before being transferred to a 0.2% MS-222 (tricaine methanesulfonate) solution at pH 7.0, where an overdose of the anesthetic was used to induce deep anesthesia and euthanasia [53,54]. Once the animals lost all reflex activity, including complete cessation of opercular movement, spinal dislocation was immediately performed to ensure rapid and painless destruction of the central nervous system, preventing sensory recovery and ensuring that sample collection could be conducted without causing pain. The animals were then transferred to a cooled dissection board lined with absorbent paper towels. Kidney tissue was dissected following the method of Gary F. Gerlach [55,56]. After being washed with saline, the tissues were promptly transferred into centrifuge tubes containing lysis buffer for subsequent analysis.

This study was conducted under the guidance of the Animal Experimentation Ethics Committee of Sichuan University (IACUC).

#### 4.5.2. H&E Staining of Zebrafish Renal Tissue

Zebrafish samples from each group were anesthetized and euthanized for H&E staining. After removing head, dorsal, and tail fins, fish were washed with PBS, dried, and fixed in 4% paraformaldehyde. Tissue sections were prepared for histological analysis.

#### 4.5.3. Protein Extraction

Kidney tissue was homogenized, centrifuged (10,000 rpm, 10 min), and the supernatant collected. Protein concentration was measured using the BCA method.

### 4.6. AGEs Determination and Glycosylation Level Characterization

Protein samples were diluted with PBS to 0.1 mg/mL. Fluorescence was measured with a spectrofluorometer, using an excitation wavelength of 370 nm and emission ranges of 380–600 nm. Advanced glycation end-products (AGEs) fluorescence was specifically monitored, and PBS was used as a baseline for fluorescence correction.

### 4.7. Changes in Protein Conformation After NM Intervention

#### 4.7.1. Secondary Structure Analysis by Circular Dichroism (CD)

Protein samples were prepared as described in 2.5. CD spectra were recorded from 200–260 nm with a scan rate of 100 nm/min and a 0.1 cm cuvette. Data were expressed as residual ellipticity (θ).

#### 4.7.2. Tertiary Structure Analysis

Fluorescence measurements were taken for each protein sample using a spectrofluorometer with excitation at 280 nm and emission between 280 and 450 nm. PBS was used for baseline correction.

The hydrophobicity of proteins was measured with the fluorescent probe ANS. Protein solutions (0.1 mg/mL) were mixed with ANS (final concentration 10 μM) and incubated at room temperature for 30 min. Fluorescence was measured from 400 to 700 nm with excitation at 380 nm.

### 4.8. Effect of NM on Glycosylation-Induced Protein Aggregation

#### 4.8.1. SDS-PAGE to Detect Protein Aggregation Residues

Protein samples were centrifuged (10,000 rpm, 15 min), and 20 μL of the supernatant was mixed with 5 μL of sample buffer. Samples were heated at 95 °C for 5 min and then cooled. SDS-PAGE was performed using 10% gels with 80 V for stacking and 120 V for separation. After staining with Coomassie Brilliant Blue R-250, images were captured with a gel imaging system and analyzed for protein aggregation.

#### 4.8.2. ThT Fluorescent Probe to Investigate Amyloid Changes in Protein

Protein samples were prepared as in 2.5. ThT (final concentration 40 μM) was added, and the mixture was incubated for 30 min. Fluorescence intensity was measured with excitation at 440 nm and emission at 482 nm.

#### 4.8.3. Inverted Fluorescence Microscopy for Protein Aggregates

Protein samples were stained with Nile Red (100 μM) for 30 min, loaded onto slides, and observed under an inverted fluorescence microscope.

### 4.9. NM–Protein Interaction by Fluorescence Quenching

Protein samples (0.1 mg/mL) were incubated with NM at concentrations ranging from 0 to 100 μM at 25 °C and 37 °C for 3 h. Fluorescence intensity was measured with excitation at 280 nm and emission from 280 to 450 nm.

Fluorescence quenching analysis was used to study the interaction between NM and protein, characterized by the Stern–Volmer Equation (2) [57]:

*F*_0_/*F* = 1 + *k*_q_*τ*_0_ [Q] = 1 + *K*_sv_ [Q] (2)

where *F*_0_ and *F* represent fluorescence intensities before and after NM addition, *k*_q_ is the quenching rate constant, *τ*0 is the protein’s fluorescence lifetime (~1 × 10^−8^ s), [Q] is the NM concentration, and *K*_sv_ is the Stern–Volmer quenching constant [58].

Additionally, the binding constant (*K_b_*) and number of binding sites (*n*) between protein and NM were determined using the Scatchard Equation (3):

log [(*F*_0_ − *F*)/*F*] = log *K*_b_ + *n* log[Q] (3)

The interaction between small molecules and proteins involves various forces, including van der Waals forces, hydrogen bonds, electrostatic interactions, and hydrophobic forces. Thermodynamic parameters (Δ*H*, Δ*S*, and Δ*G*) reveal the nature of these interactions [59]. These constants were calculated using the Van’t Hoff Equation (4) and Gibbs free energy in Equation (5). By analyzing the entropy change using Equation (6) and considering both Δ*H* and Δ*S*, the contributions of various forces, such as hydrophobic interactions, van der Waals forces, and hydrogen bonding, to the drug–protein binding process can be further elucidated [60,61].

ln (*K*_2_/*K*_1_) = Δ*H* (*T*_2_ − *T*_1_)/R *T*_1_*T*_2_ (4)

Δ*G* = −RTln*K* (5)

Δ*S* = (Δ*H* − Δ*G*)/*T* (6)

where *K* is the binding constant, *R* is the gas constant (8.314 J·mol^−1^·K^−1^), and *T* is the absolute temperature.

### 4.10. Statistical Analysis

Data were expressed as mean ± SD. Group differences were analyzed by independent samples *t*-test. Statistical significance was defined as * *p* < 0.05, ** *p* < 0.01, *** *p* < 0.001, and **** *p* < 0.0001.

## Figures and Tables

**Table 1:** Effects of NM on protein levels in the normal and model groups.

Group	*T*	*K_SV_*	*K*_b_	*K_q_*	△*G*	△*H*	△*S*
	(K)	(L•mol^−1^)	(L•mol^−1^)	(L•mol^−1^•s ^−1^)	(kJ•mol^−1^)	(kJ•mol^−1^)	(J•mol^−1^•K^−1^)
Normal	298	0.1304	145,077.48	1.30 × 10^13^	−29.45	144,795.47	584.70
	310	0.1279	1,393,477.63	1.28 × 10^13^	−36.46		584.70
Model	298	0.1448	192,752.49	1.45 × 10^13^	−30.15	−40.53	237.17
	310	0.1462	363,078.05	1.46 × 10^13^	−32.99		237.17

## Data Availability

The raw/processed data required to reproduce these findings cannot be shared at this time as the data also form part of an ongoing study.
